# Supplementary material for: Effectiveness and Safety of Ayurvedic Medicines in Type 2 Diabetes Mellitus Management: A Systematic Review and Meta-Analysis
Source: Front Pharmacol. 2022 Jun 8;13:821810. doi: 10.3389/fphar.2022.821810 (PMC9213670; doi:10.3389/fphar.2022.821810)
Supplement: Supplementary file 1 [file DataSheet1.zip › Supplementary Material/Forest and Funnel Plots/Nigella sativa/BMI.pdf]

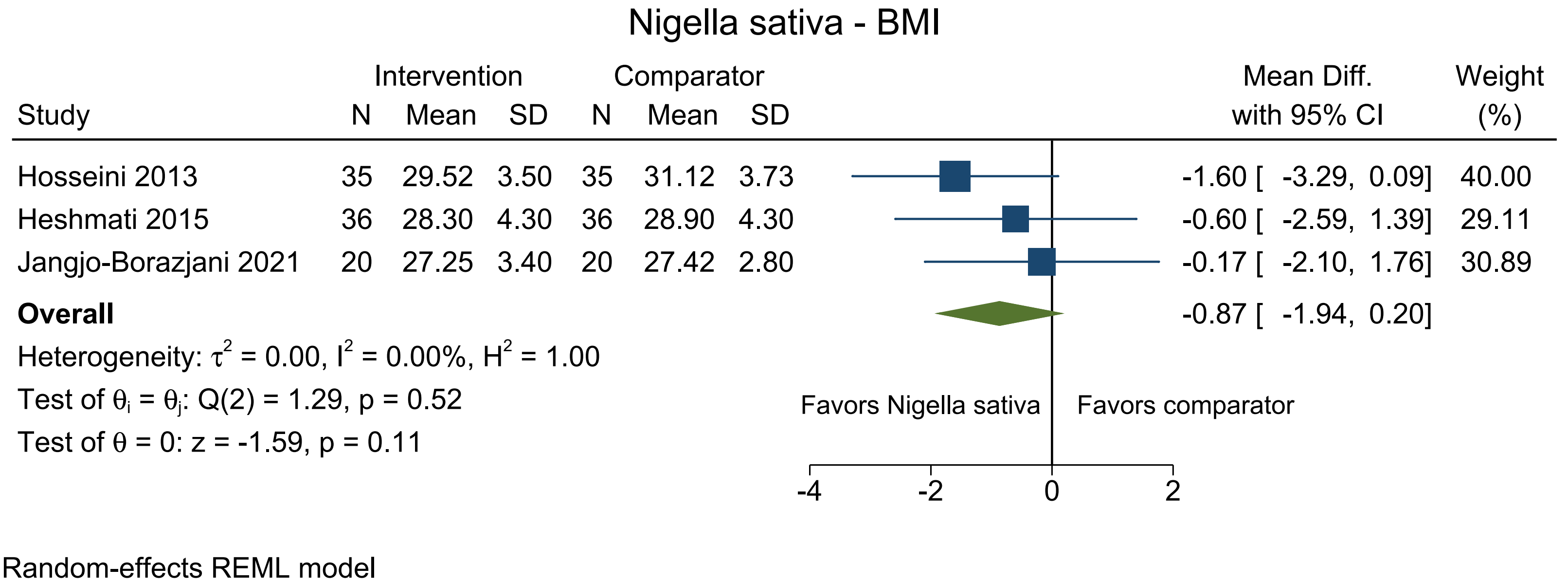
Nigella sativa - BMI
Intervention
Comparator
Study
N
Mean
SD
N
Mean
SD
Mean Diff. with 95% CI
Weight (%)
Hosseini 2013 35 29.52 3.50 35 31.12 3.73 -1.60 [-3.29, 0.09] 40.00
Heshmati 2015 36 28.30 4.30 36 28.90 4.30 -0.60 [-2.59, 1.39] 29.11
Jangjo-Borazjani 2021 20 27.25 3.40 20 27.42 2.80 -0.17 [-2.10, 1.76] 30.89
Overall -0.87 [-1.94, 0.20]
Heterogeneity: τ^2 = 0.00, I^2 = 0.00%, H^2 = 1.00
Test of $\theta_i = \theta_j$: Q(2) = 1.29, p = 0.52
Test of θ = 0: z = -1.59, p = 0.11
Favors Nigella sativa
Favors comparator
-4
-2
0
2
Random-effects REML model
